# Use of Molecular Methods for the Rapid Mass Detection of* Schistosoma mansoni* (Platyhelminthes: Trematoda) in* Biomphalaria* spp. (Gastropoda: Planorbidae)

**DOI:** 10.1155/2017/8628971

**Published:** 2017-01-26

**Authors:** Roberta Lima Caldeira, Liana Konovaloffi Jannotti-Passos, Omar Dos Santos Carvalho

**Affiliations:** ^1^Laboratório de Helmintologia e Malacologia Médica, Centro de Pesquisas René Rachou/Fiocruz, Av. Augusto de Lima, 1715 Belo Horizonte, MG, Brazil; ^2^Moluscário Lobato Paraense, Centro de Pesquisas René Rachou/Fiocruz, Av. Augusto de Lima, 1715 Belo Horizonte, MG, Brazil

## Abstract

The low stringency-polymerase chain reaction (LS-PCR) and loop-mediated isothermal amplification (LAMP) assays were used to detect the presence of* S. mansoni* DNA in (1) Brazilian intermediate hosts (*Biomphalaria glabrata*,* B. straminea,* and* B. tenagophila*) with patent* S. mansoni* infections, (2)* B. glabrata* snails with prepatent* S. mansoni* infections, (3) various mixtures of infected and noninfected snails; and (4) snails infected with other trematode species. The assays showed high sensitivity and specificity and could detect* S. mansoni* DNA when one positive snail was included in a pool of 1,000 negative specimens of* Biomphalaria*. These molecular approaches can provide a low-cost, effective, and rapid method for detecting the presence of* S. mansoni* in pooled samples of field-collected* Biomphalaria*. These assays should aid mapping of transmission sites in endemic areas, especially in low prevalence regions and improve schistosomiasis surveillance. It will be a useful tool to monitor low infection rates of snails in areas where control interventions are leading towards the elimination of schistosomiasis.

## 1. Introduction

In Africa and Neotropical region there are several* Biomphalaria* (Gastropoda: Planorbidae) species that act as the intermediate snail hosts of* Schistosoma mansoni* Sambon, 1907. In Brazil, there are 11 species but only* Biomphalaria glabrata* (Say, 1818),* Biomphalaria tenagophila* (Orbigny, 1835), and* Biomphalaria straminea* (Dunker, 1848) have been found naturally infected by this trematode. The diagnosis is routinely performed through detection of* Schistosoma mansoni* cercariae by means of artificial light exposure or by squeezing snails between glass slides and observing larval stages of the parasite under the microscope.

However, these diagnostic methods are limited and may not detect early prepatent infections in snails. In addition many field-collected snails may be infected with different trematode species and sometimes snails may die before reaching the laboratory. Therefore, molecular techniques have been developed to detect this parasite within snails and in the environment. The polymerase chain reaction (PCR) assays for amplification of the Sm1-7 repeated sequence of* S. mansoni* [1] and the low stringency-PCR (LS-PCR) for amplification of tandem DNA region of* S. mansoni* [2] have been used to detect parasites during the prepatent period. Moreover, Hamburger et al. [3] used the PCR technique to detect* S. mansoni* DNA in a pool of molluscs as well as in water after filtering using “Browne Capsule” [4].

The loop-mediated isothermal amplification (LAMP) assay was used to detect* S. mansoni* and* Schistosoma haematobium* in molluscs, one day after exposure to miracidia [5]. Later, this technique was applicable in labs with limited facilities, since the procedure does not require a thermocycler and, sometimes, it does not demand electrophoresis apparatus [6]. LAMP is a technique developed by Notomi et al. [7] to detect simple DNA, and it is sensitive and fast, using* Bst* DNA polymerase enzyme to DNA amplification in a constant temperature, which varies from 60 to 65°C. This technique was initially described using two pairs of primers, one outer pair (F3 and B3) and another inner pair (FIP and BIP), which amplify six DNA different regions, making this technique very specific. In 2002, Nagamine et al. [8] added two new primers named loop primers, which target new regions to star DNA amplification, providing a more specific and rapid method.

In this study, the LAMP assay was used with loop primers for mass detection of* S. mansoni* in* Biomphalaria* spp. Simultaneously, the* S. mansoni* DNA was amplified by LS-PCR, a technique used with success in our lab to detect the parasite in molluscs. The work aimed at establishing if these techniques could be used to monitor infected molluscs in endemic areas in order to predict the risk and dissemination of the disease, as well as assess the value of the approach for schistosomiasis control and elimination program when interruption of transmission needs to be verified.

## 2. Material and Methods

*Biomphalaria glabrata*,* B. tenagophila,* and* B. straminea* molluscs and AL, SJ, and LE strains of* S. mansoni* used in this study were maintained and raised in the “Lobato Paraense” Mollusc Rearing of René Rachou Research Center, CPqRR/Fiocruz, in Belo Horizonte, MG, Brazil. The LE strain was isolated, in 1968, from a patient residing in Belo Horizonte, MG (Brazil). The SJ strain was isolated, in 1975, from naturally infected snails from São José dos Campos, São Paulo (Brazil). The AL strain was isolated in 1980 from* B. glabrata* originated from Alagoas state (Brazil).

### 2.1. Samples

#### 2.1.1. Trematode Groups

The pools of cercariae used were (1) LE, SJ, and AL strains of* S. mansoni*, (2)* Cercaria macrogranulosa* (simple tail), (3)* Cercaria caratinguensis* (longifurcate tail), and (4)* Cercaria ocellifera* (brevifurcate tail). Trematodes from groups 2–4 were obtained from field snails* Biomphalaria* from Minas Gerais, Brazil.

#### 2.1.2. Molluscs

(A)* Biomphalaria glabrata*,* B. tenagophila*, and* B. straminea* were individually used in different conditions: (1) patent infections shedding* S. mansoni* cercariae (experimental infection with LE, SJ, and AL, resp.); (2) during the prepatent period (11 days after experimental infection); and (3) noninfected (not exposed to infection).

(B)* S. mansoni*-infected* B. glabrata* were dead for eight weeks. In summary, two specimens (10–12 mm), shedding* S. mansoni* cercariae (experimental infection with LE strain: 10 miracidia/mollusc), were recovered from the aquarium and kept in Petri dishes at room temperature for eight weeks; after this period the soft tissues were decomposed. Then, the empty shells and dried tissues were used for DNA extraction [9].

(C) Field-collected* B. glabrata* were from Ribeirão das Neves/Minas Gerais (Brazil), shedding* S. mansoni* cercariae.

(D)* Mollusc Pools*. This group was performed in triplicate in order to check the reproducibility of the techniques:* B. glabrata* specimens were exposed to 8 miracidia/mollusc from LE strain. Two snail samples during the prepatent period (11 days after exposure) were squashed and sporocysts were found. The squashed molluscs were added to negative* B. glabrata* (different ages) pools, separately, as follows: 1 positive snail in a pool of 20 negative (1 : 20) snails (weighing nearly 8 g) and 1 : 300 (104 g).

Four* B. glabrata* shedding cercariae (patent infections) were added separately to pools of negative* B. glabrata* (different ages), as follows: 1 : 20 (8 g); 1 : 300 (104 g); 1 : 500 (204 g); and 1 : 1000 (390 g).

*B. straminea* specimens were exposed to 20 miracidia/mollusc from the AL strain. One of these, in prepatent period (11 days after exposure), was squashed and sporocysts were observed. This squashed mollusc was added to a pool of negative* B. straminea* (different ages), as follows: 1 : 100 (12 g).

One* B. straminea* snail shedding cercariae was added to a pool of negative* B. straminea* (different ages) as follows: 1 : 100 (12 g).

#### 2.1.3. Molluscs Pool Preparation for DNA Extraction

The pools of snails were mashed in one container and submitted to the pepsin digestion protocol by Wallace & Rosen [10] and followed by sedimentation method of Baermann adapted [11]. After sedimentation, the material was centrifuged at 12,000*g* for 10 min and the precipitate was cryopreserved (−70°C) prior to DNA extraction.

### 2.2. DNA Extraction

In the individual mollusc group the extraction process was performed using all the body. Cercariae were pooled prior to DNA extraction. In the groups involving pooled snails, a small part of precipitated cryopreserved tissue (approximately 20 mg) was used as template for the DNA extraction.

All DNA extractions were performed using the DNA Wizard Genomic Purification kit (Promega, Madison, USA) according to the manufacturer's instructions.

### 2.3. LAMP Primer Design

The species-specific oligonucleotide primers initially used for the LAMP assay amplification were selected based on

1. mitochondrion* S. mansoni* minisatellite DNA region gene (genbank: L27240.1);
2. internal transcript spacer of rDNA gene (genbank: AY446082.1);
3. *Schistosoma mansoni* SM750 gene (genbank: M63265.1);
4. partial region of* S. mansoni* DNA mitochondrion (genbank: AF130787.1).

Thus, each sequence was submitted to LAMP Designer 1.13 software (PREMIER Biosoft International) to design the six-primer set.

### 2.4. LAMP Assays

Each LAMP reaction contained 1x ThermoPol Reaction Buffer (20 mM Tris-HCl, pH 8.8, 10 mM KCl, 10 mM (NH4)2SO4, 2 mM MgSO4, and 0.1% Triton X-100), 200 *μ*M of each dNTP, 40 pmol/*μ*L of each internal primer (FIP/BIP), 20 pmol/*μ*L of LoopF and LoopB, 5 pmol/*μ*L of each external primer (F3/B3), 0.8 M of Betaine, 6.4 U of Bst DNA polymerase, large fragment (New England Biolabs), and 2 *μ*L DNA (concentration depends on the experiment), in a final volume of 25 *μ*L. A negative control (no DNA) was included in all experiments. The mixture was incubated at 65°C for 40 min and then heated at 80°C for 10 min to terminate the reaction.

To determine the sensitivity of infection detection by LAMP, DNA was extracted from 200 cercariae from LE strain of* S. mansoni* (yielding 8.0 ng) and from one negative* B. glabrata* (yielding 13.4 ng). DNA concentrations were determined by Qubit 2.0 high sensitivity (Invitrogen) and different amounts of parasite DNA were mixed with a constant amount of negative snails in artificial infection. Thus, the DNA negative snails were diluted to 1.3 ng and mixed with 8.0 pg, 0.8 pg, 80 fg, and 8 fg of cercariae DNA and the LAMP was carried out as described above.

### 2.5. LS-PCR Assay

DNA was amplified using 0.8 units of* taq* polymerase Platinum (Invitrogen), 200 *μ*M dNTPs, 1.5 mM MgCl_2_, 50 mMKCl, 10 mM Tris-HCl, pH 8.5, 5 pmol of each primer (ER 5′ ACCTACCGTACTATGACG and EF 5′ GGTTTCTTAGTGTTATAGCC) [12], and 1 *μ*L DNA (concentration depends on the experiment) in a final volume of 10 *μ*L. The pair of primers used in these reactions was designed to amplify across adjacent in tandem minisatellite units from* S. mansoni* mtDNA [12]. A negative control (no DNA) was included in all experiments. DNA was amplified in thermocycler, using 35 cycles of amplification under the following conditions: an initial denaturing step at 95°C for 3 min, annealing 40°C for 1 min, and extension at 72°C for 1 min. For the following 34 cycles, the denaturing step was reduced to 45 sec. A final extension step (72°C) was added at the last 5 min of reaction.

The sensitivity LS-PCR was 0.1 pg* S. mansoni* DNA in 0.1 ng of DNA snail, as showed by Jannotti-Passos et al. [2].

### 2.6. Amplicon Detection

#### 2.6.1. Gel Electrophoresis to LAMP and LS-PCR

Three microliters of LAMP products was examined on a 6% polyacrylamide gel and stained with silver. The remaining was used in the colorimetric detection to LAMP.

#### 2.6.2. Colorimetric Detection to LAMP

DNA intercalating dye SYBR Green I [5] and SYBR Safe DNA gel stain (both by Invitrogen) was added to the solution after the reaction was completed. When the LAMP reaction is positive the color of the reaction solution changed from orange to yellow/green in the presence of LAMP amplicon.

## 3. Results

### 3.1. LAMP Primer Design

Each set of six primers was submitted to LAMP reaction and only one set was specific to amplify* S. mansoni* DNA. This set of primers was from ITS rDNA of* S. mansoni* and were named:

LAMPsmITS_F3 (5′ACGCACATTAAGTCGTGG 3′); LAMPsmITS_B3 (5′AACCAGAGACAAGATCAAGTG 3′); LAMPsmITS_FIP (5′ACCGCAGCATCTCAATCAAGTCAGAGGCTCCGTCCTAAT 3′); LAMPsmITS_BIP (5′TTGTGCTCGAGTCGTGGCGCATACGATAGGTGCGAAT 3′); LAMPsmITS_LoopF (5′ATCTAGACCAGACTAGGCTGT3′); LAMPsmITS_LoopB (5′GACATTATACACGCTCGGGAT 3′). Thus, this set was used in all LAMP assay.

### 3.2. LS-PCR and LAMP Assays

**Figure 1:**
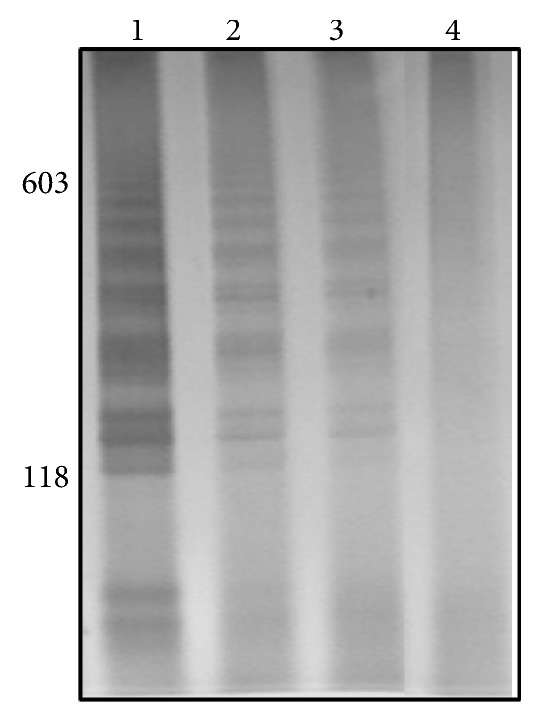
Silver-stained 6% polyacrylamide gel showing LAMP products from sensitivity test. Lane 1: DNA negative snails (1.3 ng) mixed with 8.0 pg from cercariae DNA, Lane 2: DNA negative snails (1.3 ng) mixed with 0.8 pg of cercariae DNA, Lane 3: DNA negative snails (1.3 ng) mixed with 80 fg of cercariae DNA, and Lane 4: DNA negative snails (1.3 ng) mixed with 8 fg of cercariae DNA. Molecular-size markers are shown on the left of the gel.

**Figure 2:**
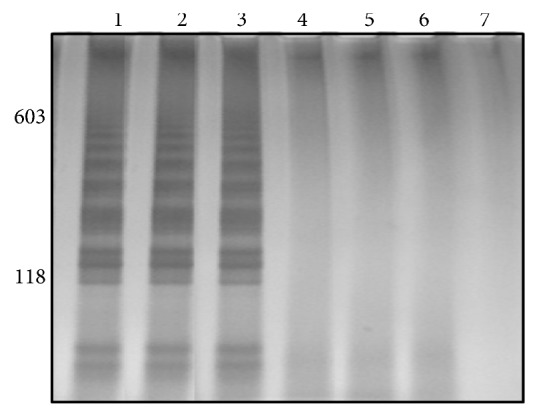
Silver-stained 6% polyacrylamide gel showing LAMP products from specificity test. Lane 1: DNA from* Schistosoma mansoni* cercariae LE strain; Lane 2: DNA from* S. mansoni* cercariae SJ strain; Lane 3: DNA from* S. mansoni* cercariae AL strain; Lane 4: macrogranulosa cercariae; Lane 5: caratinguensis cercariae; Lane 6: ocelifera cercariae; Lane 7: negative control. Molecular-size markers are shown on the left of the gel.

The LAMP sensitivity was evaluated with a limit of detection observed at 70 fg of DNA (Figure 1). The specificity LAMP assay was tested by amplification of DNA from* S. mansoni* cercariae of the strains LE, SJ, and AL, while the amplification of DNA from cercariae* C. macrogranulosa*,* C. caratinguensis,* and* C. ocellifera* were not detectable (Figure 2). The specificity and sensitivity of infection detection by LS-PCR have been made previously by Jannotti-Passos et al. [2].

**Figure 3:**
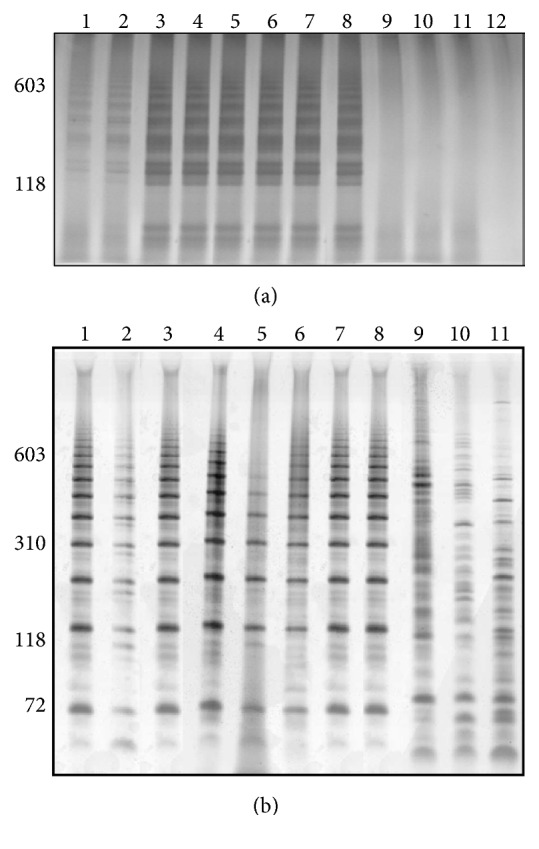
Silver-stained 6% polyacrylamide gel showing LAMP (a) and LS-PCR (b) products. Lane 1:* Biomphalaria glabrata* shedding* Schistosoma mansoni* cercariae; Lane 2:* Biomphalaria tenagophila* shedding* S. mansoni* cercariae; Lane 3:* Biomphalaria straminea* shedding* S. mansoni* cercariae; Lane 4: infected* B. glabrata* in prepatent period; Lane 5: infected* B. tenagophila* in prepatent period; Lane 6: infected* B. straminea* in prepatent period; Lane 7:* S. mansoni*-infected* B. glabrata* dead for eight weeks; Lane 8: field-collected* B. glabrata* shedding* S. mansoni* cercariae from Ribeirão das Neves/MG (Brazil); Lane 9: negative* B. glabrata*; Lane 10: negative* B. tenagophila* Lane 11: negative* B. straminea*; and Lane 12: negative control (only in LAMP). Molecular-size markers are shown on the left of the gel.

LAMP and LS-PCR amplifications presented characteristic profile to* S. mansoni* in* B. glabrata*,* B. tenagophila,* and* B. straminea* shedding* S. mansoni* cercariae; infected* B. glabrata*,* B. tenagophila,* and* B. straminea* in prepatent period;* S. mansoni*-infected* B. glabrata* dead for eight weeks and* B. glabrata* shedding* S. mansoni* cercariae from Ribeirão das Neves/MG (Brazil) (Figures 3(a) and 3(b)).

**Figure 4:**
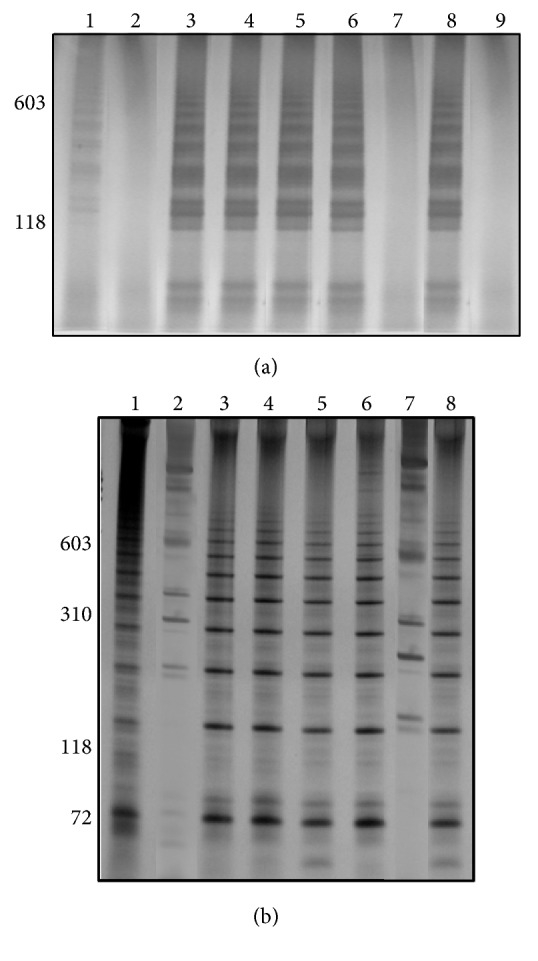
Silver-stained 6% polyacrylamide gel showing LAMP (a) and LS-PCR (b) from molluscs pool. Lanes 1 and 2: infected* Biomphalaria glabrata* in prepatent period: 1 positive snail in a pool of 20 negative snails and 1 : 300; Lanes from 3 to 6: infected* B. glabrata* shedding cercariae: 1 : 20; 1 : 300; 1 : 500, and 1 : 1000; Lane 7: infected* Biomphalaria straminea* in prepatent period: 1 : 100; Lane 8: infected* B. straminea* shedding cercariae; Lane 9: negative control (only in LAMP). Molecular-size markers are shown on the left of the gel.

For molluscs pool amplifications LAMP and LS-PCR assays presented characteristic profile to* S. mansoni* in all amplifications, except in pool of 300 negative snails and one* B. glabrata* in prepatent period and in pool of 100 negative snails and one* B. straminea* in prepatent period (Figures 4(a) and 4(b), Lanes 2 and 7, resp.). All triplicates from molluscs pool group were equal (data not shown).

**Figure 5:**
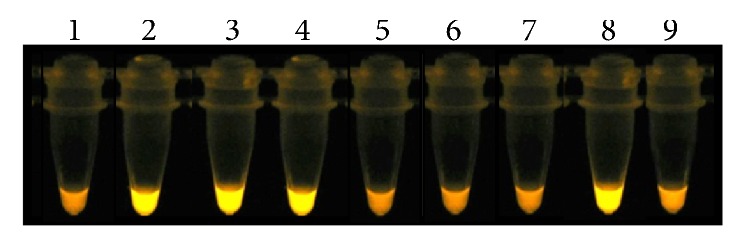
Fluorescence of LAMP products from molluscs pool using SYBR Green I staining followed by ultraviolet illumination at 320 nm. Tubes 1 and 2: infected* Biomphalaria glabrata* in prepatent period: 1 positive snail in a pool of 20 negative snails and 1 : 300; Tubes from 3 to 6: infected* B. glabrata* shedding cercariae: 1 : 20; 1 : 300; 1 : 500; and 1 : 1000; Tube 7: infected* Biomphalaria straminea* in prepatent period: 1 : 100; Tube 8: infected* B. straminea* shedding cercariae; Tube 9: negative control.

The amplicons of LAMP were observed by gel (Figures 1–4) and colorimetrics methods. However, the colorimetric detection (SYBR Green I and SYBR Safe DNA) was not reproducible and caused concern about false-positives and false-negatives.  Figure 5 illustrates the inconsistency in the interpretation of results after staining with SYBR Green I and exposure to ultraviolet light. The samples are the same as those applied to the gel of Figure 4.

## 4. Discussion

The possibility of diagnosing* S. mansoni* in pooled snails will accelerate the detection of new snail habitats and could assist schistosomiasis control programs by highlighting freshwater bodies associated with schistosomiasis transmission. Thus, the main contribution of this work was the use of molecular assays for DNA detection of parasite that showed that it was possible to detect one infected snail in a pool of 1000 negative* B. glabrata*. LS-PCR and LAMP assays were sensitive and specific to detection. The LAMP assay saves time and does not require an expensive thermocycler since all reactions can be performed at a constant temperature [13]. In same experiments colorimetric assays are used as dyes that are added to the solutions before or after LAMP reaction. Later, these authors [13] compared different dyes to analyze the sensitivity of LAMP assay, including hydroxy naphthol blue (HNB), calcein, and SYBR Green: the first two being added before the reaction and the last one after the reaction. These authors observed that the HNB sensitivity was equivalent to SYBR Green. But the tubes with HNB dye were not required to be opened to determine the results, reducing the risk of cross-contamination. LAMP technique was used to detect* Leishmania* DNA directly from infected sand fly extract, using colorimetric malachite green (MG) by naked eye visualization [14]. However, the MG and HNB dyes are difficult to discern by eye due to the subtleness in the difference between positive and negative results [14–17]. The main disadvantage of using SYBR Green I is the possibility of cross-contamination. Indeed, Abbasi et al. [5] observed that the detection by SYBR Green I was not possible in the case of examining snails because uninfected snails also showed positive fluorescent signals given the amount of snail DNA present in the reaction mixture. Similarly, when we used SYBR Green I and SYBR Safe DNA in the LAMP reactions from* S. mansoni*-infected* Biomphalaria*, interpretation problems were encountered with the possibility of false-positives (see Figure 5). Therefore our preference was to run the LAMP products on 6% polyacrylamide gels and to use silver staining.

LAMP assays have been used for the detection of different species of* Schistosoma*, either in intermediate or in definitive hosts [5, 18–20]. Hamburger et al. [6] adapted the LAMP tool for application in field laboratories in endemic areas for* S. haematobium* and* S. mansoni*. These field laboratories lacked the facility and expert teams in molecular biology. They demonstrated that a DNA amplification tool suitable for field laboratories ensures a broader examination of LAMP efficacy in various epidemiologic settings. LAMP assay was used to detect* Schistosoma japonicum* infection in pooled samples of field-collected* Oncomelania* in China and they constructed a transmission of schistosomiasis risk map using ArcMap, based on the positive proportion, which will be a surveillance and response strategies guide in high risk areas [21]. Kumagai et al. [22] reported that LAMP and conventional PCR could amplify* S. japonicum* DNA from a group of 100 normal snails artificially mixed with one infected snail; consequently, the detection from pooled samples has opened the door for large-scale low-cost screening.

Regarding costs and time per reaction, the LAMP is approximately half the price and the time compared with LS-PCR. If the cost of thermocyclers for DNA amplification is also included, LAMP becomes comparatively even cheaper given that it can be performed using only a heat block. On the other hand, samples without* S. mansoni* DNA presence show no specific amplicons when LS-PCR technique is used, providing a more accurate result, since it works as an internal control.

The principal contribution of this study was to detect one* B. glabrata* infected with* S. mansoni* in a pool of 1000 negative snails, regardless of the technique used. This strategy is interesting to provide a low-cost, rapid, and effective method to detect* S. mansoni* DNA in field-collected* Biomphalaria*. The next step is to validate this strategy using snails collected from the field and to carry out mapping of transmission sites. This assay might improve schistosomiasis surveillance by providing a much needed tool to monitor infections in snails especially in low transmission areas.
